# Person-Centred Care in Association with Psychological Well-Being of Older Adults

**DOI:** 10.3390/healthcare13121379

**Published:** 2025-06-09

**Authors:** Mateja Lorber, Nataša Mlinar Reljić, Brendan McCormack, Sergej Kmetec

**Affiliations:** 1 Faculty of Health Sciences, University of Maribor, 2000 Maribor, Slovenia; natasa.mlinar@um.si (N.M.R.); sergej.kmetec1@um.si (S.K.); 2 National Institute of Public Health, 1000 Ljubljana, Slovenia; 3 Susan Wakil School of Nursing, Faculty of Medicine and Health, University of Sydney, Camperdown Campus, Camperdown, NSW 2050, Australia; brendan.mccormack@sydney.edu.au

**Keywords:** older adults, psychological well-being, health, person-centred care

## Abstract

Background: As the global population ages, there is an increasing focus on enhancing the psychological well-being of older adults. A key strategy is person-centred care, which emphasises recognising each individual as unique, with their values, needs, and preferences. This study explored older adults’ perceptions of person-centred care and its relationship with their general health and psychological well-being. Methods: A cross-sectional study involving 632 participants aged 65 to 96 years (mean age = 73.4 ± 6.7) was conducted, comprising 435 (69%) female and 197 (31%) male participants. Of these, 57% lived in home environments, while 43% resided in retirement homes. Data were collected using a self-report questionnaire that included the General Health Questionnaire (GHQ-12), the Warwick–Edinburgh Mental Wellbeing Scale (WEMWBS), and the Person-Centred Practice Inventory for Service Users (PCPI-SU). Data analysis was performed using SPSS Statistics 28.0, and non-parametric tests such as the Kruskal–Wallis test, Mann–Whitney U test, and Spearman’s correlation were used. Results: Older adults who reported more frequent contact with relatives or loved ones (daily or several times per week) and those residing in home environments perceived significantly higher levels of person-centred care compared to those with less frequent contact and those living in retirement homes (*p* < 0.001 for both variables). General health varied significantly according to gender (*p* = 0.009), level of education (*p* < 0.001), and living environment (*p* = 0.004), while psychological well-being among older adults showed significant differences based solely on their level of education (*p* < 0.001). Regression analysis revealed that person-centred care (*p* = 0.017) and monthly income (*p* < 0.001) were significantly associated with the psychological well-being of older adults, independent of their living environment. Conclusions: The findings suggest that person-centred care and monthly income significantly predict psychological well-being among older adults. Differences in perceived care and health outcomes were also observed based on gender, education level, and living environment. These results underscore the importance of promoting person-centred care practices, particularly in retirement home settings, to enhance psychological well-being in older adult populations.

## 1. Introduction

As the global population ages, there is a growing emphasis on improving the psychological well-being of older adults. According to the World Health Organisation (WHO), the number of people aged 60 and older is projected to reach 2.1 billion by 2050, nearly doubling from 1 billion in 2020 [1]. A rise in age-related health challenges, including chronic non-communicable diseases, cognitive decline, and mental health disorders such as depression and anxiety, accompanies this demographic shift. In the European region, approximately 20% of older adults are affected by mental health conditions, with depression ranking among the leading causes of disability [2,3].

Regionally, Slovenia mirrors these global trends. Data from the Statistical Office of the Republic of Slovenia (SURS) indicate that individuals aged 65 years and above accounted for 21.4% of the total population in 2023 [4]. Additionally, national health reports highlight increasing trends in chronic illness and psychological distress among this age group, emphasising the need for responsive and individualised care approaches [5].

One widely adopted approach in geriatric care is person-centred care, which focuses on recognising individuals as unique persons with distinct values, needs, and preferences. This model of care contrasts with traditional medical approaches by prioritising autonomy, dignity, and personal choice, ultimately leading to better psychological well-being among older adults [6,7]. Psychological well-being in older adults refers to a positive state of mental health that includes emotional balance, life satisfaction, a sense of purpose, and resilience against stress and depression [8]. Older adults are particularly vulnerable to psychological distress due to factors such as social isolation, cognitive decline, chronic illness, and loss of independence [9]. Addressing these factors through person-centred care interventions can significantly improve their well-being. Several studies underscore that the psychological well-being of older adults is influenced by factors such as gender, education, income, and living arrangements. For instance, Liu & Su [10] identified monthly income and education level as key predictors of healthy ageing. At the same time, Kim & Park [11] found that person-centred care was especially effective in enhancing well-being among older adults with higher health literacy.

Person-centred care is an approach that places the individual at the core of healthcare decision-making. It involves tailoring services to meet each person’s needs, preferences, and life experiences rather than applying a standardised treatment model. Key principles of person-centred care include [12] respect for individuality, which means recognising each older adult’s personal history, values, and experiences; dignity and autonomy, relating to encouraging older adults to participate in decisions about their care; meaningful relationships, including strengthening social connections between caregivers and older adults; holistic approach, covering addressing not only physical health but also emotional, social, and psychological needs. Older adults frequently experience multiple, complex health conditions, positioning them as prime candidates to benefit from person-centred care [13]. Empirical studies have demonstrated that person-centred care is associated with improved psychological and physical health outcomes among older adults. For example, Sjögren et al. [14] found a positive relationship between perceived person-centredness and resident well-being in dementia care units, while Edvardsson et al. [15] reported that person-centred interventions improved life satisfaction and engagement among people with severe Alzheimer’s disease.

The World Health Organisation (WHO) defines person-centred care as an approach that considers individuals holistically, recognising their varied needs and goals within the caregiving process [16]. The American Geriatrics Society emphasises that person-centred care necessitates individuals’ willingness to articulate their values and preferences [17]. McCormack’s framework for person-centred practice highlights “best practices” across four dimensions: prerequisites (focusing on the caregiver’s attributes), the care environment, person-centred processes, and outcomes such as satisfaction and well-being [18]. While broad agreement exists on the components of person-centred care, applying these principles in real-world settings remains challenging [19]. In recent years, person-centred care has gained significant attention, prompting the WHO to advocate for person-centred policies that tackle the complex challenges people encounter in their communities [20].

Research highlights the positive association between person-centred care and the psychological well-being of older adults. Studies suggest that person-centred care enhances general health and improves functions, well-being, and quality of life [14,21,22]. Person-centred care fosters meaningful engagement in activities, improving life satisfaction [19]. Providing individualised attention and emotional support lowers the risk of mental health issues [23]. Encouraging participation in social activities reduces loneliness and enhances well-being [24]. Personalised cognitive stimulation activities help slow cognitive decline and promote a sense of competence [11]. Despite its benefits, implementing person-centred care in healthcare settings comes with challenges, such as staff training and attitudes [25], resource limitations because adequate staffing and time are necessary to provide personalised care, and institutional barriers because traditional medical models may conflict with person-centred care approaches, requiring systemic changes.

Despite growing awareness of the importance of psychological well-being in ageing populations, traditional healthcare models often remain focused on clinical outcomes and standardised treatments, overlooking the complex, individualised needs of older adults. As chronic diseases, cognitive decline, and social isolation increasingly affect this demographic, there is a pressing need to adopt approaches that prioritise dignity, autonomy, and holistic support. Social support also plays a critical role in the psychological well-being of older adults. Brooker & Latham [12] emphasised that meaningful relationships, particularly frequent contact with loved ones, are central to person-centred care and are positively associated with improved mental health outcomes. Person-centred care offers a framework that aligns healthcare delivery with older adults’ lived experiences and preferences, potentially improving health outcomes and overall life satisfaction. However, empirical evidence on the perceived quality and effectiveness such care remains limited, particularly across different living environments. This study addresses this gap by examining older adults’ perceptions of person-centred care and its associations with general health and psychological well-being. It provides insights critical for developing targeted, responsive interventions.

We aimed to explore the perception of person-centred care among older adults and examine its relations with general health and psychological well-being. Additionally, this study investigates differences in gender, living environments, number of contacts with relatives or loved ones, and monthly income related to psychological well-being. These elements are critical for understanding the complex experiences of older adults and developing tailored interventions. The association of living environments is especially significant, as older adults in retirement homes might face distinct social dynamics, healthcare access, and physical challenges compared to home environments.

## 2. Materials and Methods

### 2.1. Design

This quantitative cross-sectional study examined the relationships between person-centred care provided and the psychological well-being of older adults. The research rigorously followed the STROBE (Strengthening the Reporting of Observational Studies in Epidemiology) guidelines to guarantee the reliability and clarity of the findings.

### 2.2. Study Sites/Settings and Populations

A purposive sampling method was employed to select participants who fulfilled specific criteria pertinent to this study’s goals, focusing particularly on older adults capable of providing dependable self-reported data. Inclusion criteria required participants to be 65 years or older and capable of providing reliable self-reported data. Exclusion criteria included individuals with a diagnosed mental disorder, as such conditions could confound the study variables related to psychological well-being and perceived care quality. To reduce biases in sampling and recruitment, efforts were made to include participants from various healthcare settings across different regions, including primary care centres and retirement homes.

Using the Cochran formula [26] and based on the population of older adults aged 65 and above (*n* = 453,708), we calculated that a sample size of 399 older adults would be necessary. One thousand questionnaires were selectively distributed to eligible participants identified through purposive sampling, based on inclusion and exclusion criteria. Of these, 724 were returned, yielding a response rate of 63%. Ninety-two questionnaires were disqualified due to incomplete responses (less than 70%), resulting in a final sample size of 632 older adults.

### 2.3. Participants

Six hundred thirty-two older adults participated in this study, aged 65 to 96 years (mean = 73.4, SD = 6.7; 95%CI: 72.9–74.0). The sample comprised 435 females (69%) and 197 males (31%). Regarding living arrangements, 359 participants (57%) lived in home environments, while 273 (43%) resided in retirement homes. Educational attainment varied: 110 participants (18%) had completed elementary school, 396 (63%) had secondary education, 110 (17%) held an undergraduate degree, and 16 (3%) had completed postgraduate studies.

### 2.4. Study Toll/Instruments

The study participants filled out a self-report questionnaire, which included the General Health Questionnaire (GHQ-12) [27], the Warwick–Edinburgh Mental Wellbeing Scale (WEMWBS) [28], and the Person-Centred Practice Inventory for Service Users (PCPI-SU) [29]. They also provided demographic information such as gender, education level, living environment, frequency of contact with relatives or loved ones, and monthly income.

The WEMWBS, created by Tennant et al. in 2007 [28], measures the general population’s psychological well-being. It contains 14 statements rated on a five-point scale from ‘None of the time’ (1) to ‘All of the time’ (5), with scores ranging from 14 to 70; higher scores indicate better psychological well-being. This scale is highly reliable, with a Cronbach’s alpha of 0.913.

The PCPI-SU, developed by McCormack et al. [29], is a 20-item scale that measures perceptions of person-centred care across five domains: Working with the Person’s Beliefs and Values, Sharing Decision-making, Engaging Authentically, Being Sympathetically Present, and Working Holistically. This tool, based on McCormack and McCance’s frameworks [30], uses a five-point Likert scale ranging from ‘strongly disagree’ (1) to ‘strongly agree’ (5), where higher scores reflect a more favourable view of person-centred care. This scale is highly reliable, with a Cronbach’s alpha of 0.959.

The GHQ-12, developed by Goldberg and Williams [27], assesses general health severity over recent weeks through 12 items scored on a four-point Likert scale from ‘Never’ (0) to ‘Always’ (3). A lower total score indicates better general health. With this scale, Cronbach’s alpha was reported at 0.802.

### 2.5. Data Collection

Data collection occurred from July 2023 to July 2024, targeting older adults in home environments and retirement homes. Consent was obtained from both participating individuals and institutions. Healthcare organisations facilitated recruitment by connecting researchers with potential participants through primary care centres and retirement homes. Healthcare staff identified eligible older adults who met this study’s criteria, informed them about its aim, and distributed questionnaires to those interested. The research team personally handed out these questionnaires, assisting with any queries to encourage participation. Participating older adults submitted their filled-in surveys in sealed envelopes to assigned collection locations within three weeks, guaranteeing a varied sample representing different living arrangements and healthcare accessibility.

The data analysis included thorough checks for the completeness of the returned questionnaires. Responses missing critical data on key variables such as psychological well-being, general health, or person-centred care were excluded from analyses to preserve result validity. For minor missing demographic data, pairwise deletion maximised data utility without introducing bias. Ultimately, 632 completed questionnaires were analysed, reflecting a response rate of 63%.

### 2.6. Data Analysis

Data were analysed using SPSS Statistics 28.0. To assess the normality of the data, the Shapiro–Wilk test was applied to all continuous variables. The normality of continuous variables (psychological well-being, general health, and person-centred care) was assessed using the Shapiro–Wilk test and skewness and kurtosis statistics. All variables showed significant deviations from normality (*p* < 0.05) and exhibited skewness and/or kurtosis values beyond the acceptable thresholds (±2), justifying the use of non-parametric statistical methods. Consequently, the Mann–Whitney U and Kruskal–Wallis tests were used to evaluate group differences. Additionally, Spearman’s rank correlation coefficient was employed to assess associations between psychological well-being, general health, and person-centred care. Since the Shapiro–Wilk test and the evaluation of skewness and kurtosis indicated deviations from normal distribution for key continuous variables (psychological well-being, general health, and person-centred care), non-parametric tests were used. Group differences were assessed using the Kruskal–Wallis test. Post-hoc pairwise comparisons were performed using Dunn’s test with Bonferroni correction to control for multiple comparisons for variables with statistically significant Kruskal–Wallis test results. Variables included in the regression model were selected based on their theoretical relevance and significant bivariate associations with psychological well-being. A standard multiple regression using the Enter method was performed, in which all predictors were entered simultaneously to assess their independent effects. For regression analyses, gender was coded as a binary variable (0 = female, 1 = male). Multicollinearity among predictors was assessed using variance inflation factors (VIFs). A VIF value below five was considered acceptable, indicating no severe multicollinearity. All statistical tests were considered significant at a *p*-value of less than 0.05. Internal consistency of the instruments was assessed using Cronbach’s α coefficient.

## 3. Results

Statistical analyses examined differences and associations in person-centred care, general health, and psychological well-being among older adults based on gender, living environment, education level, frequency of contact with relatives or loved ones, and monthly income. Non-parametric tests were used due to non-normal data distribution, and findings are presented in the following tables.

As shown in Table 1, all five dimensions of person-centred care and the overall score were significantly lower among older adults living in retirement homes compared to those living at home (all *p* < 0.001). The largest difference was observed in the “Sharing Decision-Making” dimension (Z = 4.558, *p* < 0.001), indicating reduced involvement in care decisions among those in institutional settings.

Table 2 shows that person-centred care scores significantly differed according to living environment (Z = 3.527, *p* < 0.001) and frequency of contact with relatives or loved ones (H = 31.420, *p* < 0.001). General health scores were significantly different by gender (Z = 2.597, *p* = 0.009), level of education (H = 23.987, *p* < 0.001), and living environment (Z = 2.902, *p* = 0.004). Psychological well-being scores were significantly different according to the level of education (H = 16.324, *p* < 0.001). Post-hoc analysis using Dunn’s test with Bonferroni correction indicated that participants with postgraduate education reported significantly higher psychological well-being than those with elementary or secondary education (*p* < 0.05). Similarly, participants who had contact with relatives several times per week reported significantly higher person-centred care scores than those with contact only once a month or less (*p* < 0.05).

From Table 3, we can see that person-centred care is positively related to psychological well-being (*r_s_* = 0.122; *p* = 0.017) and several contacts with older adults’ relatives or loved ones (*r_s_* = 0.117; *p* = 0.023) and is negatively related to older adults’ age (*r_s_* = −0.127; *p* = 0.013). In the same table, we can also see that the psychological well-being of older adults is related only to person-centred care (*r_s_* = 0.122; *p* = 0.017) and monthly income (*r_s_* = 0.236; *p* < 0.001). At the same time, we can see that general health is related to age (*r_s_* = 0.107; *p* = 0.011) and monthly income (*r_s_* = 0.262; *p* < 0.001), and the number of contacts with relatives and loved ones is related to person-centred care (*r_s_* = 0.117; *p* = 0.023) and monthly income (*r_s_* = 0.134; *p* < 0.001).

With regression analysis, we found that age, gender, and person-centred care are significantly associated with general health (Table 4), and that person-centred care, monthly income, and age are significantly associated with the psychological well-being of older adults (Table 5). When we conducted a separate analysis according to the living environment, we found that for those who lived in a home environment (F = 4.704, *p* < 0.001), person-centred care (t = 2.065, *p* = 0.034) and monthly income (t = 2.572, *p* = 0.011) were lower predictors than for those who lived in a nursing home (F = 7.385, *p* < 0.001) (person-centred care (t = 2.396, *p* = 0.018); monthly income (t = 4.069, *p* < 0.001)). All VIF values for the predictors in the regression models were below 2.0, indicating no multicollinearity concerns.

## 4. Discussion

This research highlights significant outcomes from implementing person-centred care in gerontological nursing in all settings, emphasising improved psychological well-being and general health among older adults. This study’s main findings are that the participating older adults self-assessed their general health, psychological well-being, and perceived person-centred care as good or positive. The statistical analysis of various demographics, such as gender, living environment, and social interactions, sheds light on the nuances of person-centred care efficacy.

It was found that older adults perceived person-centred care at a high median level. According to the dimensions of person-centred care, the person’s beliefs and values received the highest ratings, while sharing decision-making received the lowest, though still a positive score (3.35 out of 5). Compared to the living environment, older adults who lived in a home environment rated the dimensions of sharing decision-making and working holistically the highest. Still, those living in a retirement home rated engaging authentically and being sympathetically present the highest. A notable difference exists between participants living at home versus those in retirement homes, with the home environment reporting higher scores in person-centred care metrics. This suggests that the home environment can offer a more conducive setting for implementing person-centred care principles, such as autonomy and engagement. These observations are supported by Edvardsson et al. [15], who argue that environmental familiarity and comfort significantly contribute to the effectiveness of person-centred interventions.

This research did not indicate a statistically significant difference in perceived person-centred care and psychological well-being according to gender. Males showed a slightly higher preference for delivered person-centred care, which was also evidenced by their significantly higher scores in general health but lower psychological well-being compared to females. Such findings align with previous studies [23,31], which noted gender differences in the perception of care, suggesting that tailored approaches might enhance efficacy.

The frequency of contact with relatives and loved ones is positively related to psychological well-being scores. Those interacting with relatives or loved ones daily show better general health and psychological well-being outcomes than those with less frequent interactions. This finding reinforces the importance of meaningful relationships, a core principle of person-centred care, as highlighted by Brooker & Latham [12]. The results underscore the role of social support in enhancing older adults’ general health and psychological well-being.

At the same time, we found that person-centred care was related to psychological well-being, age, and several contacts with relatives and loved ones. With regression analysis, we found that person-centred care and monthly income were significantly associated with older adults’ psychological well-being and can, together with other studied variables, explain almost 29% of the total variability of older adults’ psychological well-being. The analysis showed that person-centred care (t = 2.396, *p* = 0.018) and monthly income (t = 4.069, *p* < 0.001) are stronger predictors of psychological well-being for older adults living in retirement homes, according to those who lived in a home environment. Our results supported the results by Li-Fan et al. [10], who similarly observed a decline in general health associated with increasing age and differences related to gender. Individual-level factors such as educational background, economic status, health behaviours, and social participation emerged as strong predictors of healthy ageing and overall well-being.

Although there was no significant difference in person-centred perception based on educational level in our research, there were notable variances in general health and psychological well-being outcomes. Older adults with higher education levels, on the other hand, reported better psychological well-being, reflecting greater health literacy and proactive engagement in their care. This result supports research by Kim & Park [11] and Adams [32], which suggests that the relationship between education and health outcomes is mediated through better understanding and engagement in health-promoting behaviours.

Implementing a person-centred approach is challenging, as highlighted in the literature [25,33], due to difficulties in training staff, ensuring adequate resources, and overcoming institutional barriers. These obstacles necessitate a comprehensive approach to training and systemic changes within healthcare institutions, as detailed by Brownie & Nancarrow [25].

This study has several limitations that warrant attention. The cross-sectional design constrains our ability to determine causal relationships. Only a few existing studies compare person-centred care, psychological well-being, and general health among older adults in home environments and retirement homes. Another limitation may stem from the sampling method, which could affect the findings. An overrepresentation of one gender and location in the sample may limit the generalizability of the results to the whole population. Additionally, self-reported information might introduce social desirability bias, as respondents may amplify or understate their answers to align with perceived social norms. Moreover, the voluntary basis of participation raises questions regarding the extent to which the sample truly represents the primary attributes of the population. Additionally, this study may not have accounted for all potential confounding factors influencing psychological well-being, general health, and perceived person-centred care.

Notwithstanding certain limitations, this study highlighted the essential role of person-centred care in improving psychological well-being and general health among older adults, irrespective of their living environment. At the same time, this research provides a framework for further exploration into how person-centred care can be optimised across different settings and populations. Future studies could explore assessing the long-term benefits of person-centred care on psychological well-being and physical health, how cultural differences affect the reception and effectiveness of person-centred care, as cultural values can be significantly associated with perceptions of autonomy and individuality, and how technology, such as digital health tools, can be integrated into person-centred care to enhance connectivity and personalised care.

### Implications for Practice

The findings of this research highlight person-centred care as a fundamental approach to promoting the psychological well-being of older adults. However, effectively implementing this principle in real-world care settings, particularly in ways that meaningfully involve family and friends, requires intentional strategies, policy support, and structural investment across healthcare systems.

Clear national and regional policies are essential for implementing person-centred care principles across all healthcare contexts. They should define standard practices, set expectations for treating older adults with respect and dignity, and ensure accountability by embedding specific person-centred care requirements into existing healthcare regulations.

One of the most impactful ways to operationalise person-centred care is by adopting triadic communication models that include older adults, healthcare professionals, and informal caregivers. These models foster a holistic understanding of the older person’s context, preferences, and psychosocial needs. Triadic approaches are especially valuable in shared decision-making, allowing care teams and families to collaboratively clarify goals, navigate differing expectations, and amplify the older adult’s voice respectfully and inclusively.

To support this, health professionals should receive targeted training in communication skills. This includes techniques for navigating multi-party conversations, managing power dynamics, and maintaining the older adult as the central decision-maker. Such training should be integrated into initial curricula for medical, nursing, and allied health programs, emphasising gerontology, psychology, ethics, and communication.

Structured protocols for caregiver involvement should also be established to guide when and how family members and other support persons are included. These might, e.g., take the form of routine care planning meetings, all grounded in the consent and preferences of the older adult to safeguard autonomy and dignity.

To ensure sustainable implementation, healthcare facilities must be adequately resourced. This includes investment in staff training, environmental enhancements (e.g., accessibility, safety, comfort), and sufficient staffing levels. Additionally, interdisciplinary care teams, including doctors, nurses, social workers, and therapists, should collaborate to meet the complex biopsychosocial needs of older adults. Introducing or expanding care manager or coordinator roles can help oversee individualised care plans and maintain continuity across services.

Technology plays a critical role in enhancing person-centred care. Digital tools such as shared care platforms, electronic health records that incorporate patient preferences, and mobile apps can facilitate communication, support coordinated care, and empower older adults to express their goals and monitor their well-being, especially in home-based and community care.

Equally important is the involvement of family members and informal caregivers in the care planning process. Their participation helps ensure that decisions reflect older adults’ values and life history. Yet, person-centred care must also remain culturally sensitive and flexible, recognising that preferences for independence, privacy, and familial involvement vary greatly. Legal and regulatory frameworks should be reviewed to protect the rights of older adults and uphold their autonomy in all care decisions, including end-of-life planning.

Connecting healthcare systems with community resources can provide a broader support network. Initiatives such as local programs, social activities, and therapeutic services can reduce loneliness, foster engagement, and enhance psychological well-being. Feedback systems, incorporating input from older adults, families, and staff, are also essential for the continuous refinement and improvement of person-centred practices.

By integrating policy, workforce education, family engagement, digital innovation, environmental adaptations, and community connection, person-centred care can evolve into a practice that supports the psychological well-being and quality of life of older adults and ensures that their individual needs and preferences are respected across diverse care environments.

## 5. Conclusions

This study underscores the beneficial association of person-centred care on the psychological well-being and general health of older adults across various care settings, as the proportion of older adults rises. Furthermore, the importance of social connections is highlighted, as frequent contact with relatives correlates with improved health outcomes. Despite challenges such as the need for better training and resource allocation, the results advocate for the broader implementation of person-centred approaches, which significantly enhance the psychological well-being of older adults.

Person-centred care respects older adults’ preferences and is crucially related to their health and satisfaction. Healthcare systems need to continue adapting and innovating in person-centred care to effectively meet the ageing population’s diverse needs. Effective person-centred care requires clear national and regional policies that define standards, adapt regulations, and ensure person-centred care across healthcare settings.

## Figures and Tables

**Table 1:** Descriptive statistics of person-centred care.

Dimensions	Home (*n* = 359) $\bar{x}$ ± s	Retirement Home (*n* = 273) $\bar{x}$ ± s	Z	*p*	$\bar{x}$ ± s (95%CI)
People’s beliefs and values	3.79 ± 0.86 (1)	3.49 ± 0.91 (1)	3.265	0.001 *	3.66 ± 0.90 (3.57–3.74)
Sharing decision-making	3.60 ± 0.83 (4)	3.23 ± 0.87 (5)	4.558	<0.001 *	3.44 ± 0.86 (3.35–3.52)
Engaging authentically	3.56 ± 0.76 (5)	3.33 ± 0.83 (4)	2.587	0.010 *	3.46 ± 0.80 (3.38–3.54)
Being sympathetically present	3.64 ± 0.77 (2)	3.40 ± 0.83 (2)	2.985	0.003 *	3.53 ± 0.81 (3.45–3.61)
Working holistically	3.63 ± 0.78 (3)	3.34 ± 0.81 (3)	3.872	<0.001 *	3.50 ± 0.81 (3.42–3.58)
Person-centred care	3.66 ± 0.72	3.33 ± 0.79	3.527	<0.001 *	3.52 ± 0.67 (3.44–3.60)

Note: $\bar{x}$—mean; CI—confidence interval; S—standard deviation; Z—Mann–Whitney U; *p*—a significance value; *p* < 0.05 (*).

**Table 2:** Descriptive statistics and differences in person-centred care, general health, and psychological well-being.

Variables	Person-Centred Care 3.52 ± 0.67 95%CI: 3.44–3.60		General Health 17.93 ± 2.78 95%CI: 17.71–18.16		Psychological Well-Being 51.25 ± 8.5 95%CI: 50.34–52.17	
	*x* *±* *s*	95%CI	*x* *±* *s*	95%CI	*x* *±* *s*	95%CI
Gender	Z = 1.919; *p* = 0.055 *		Z = 2.597; *p* = 0.009 *		Z = 1.912; *p* = 0.056 *	
Male (*n* = 197)	3.46–3.74	3.61 ± 0.75	18.55 ± 2.24	18.21–18.89	49.40 ± 8.23	47.82–50.87
Female (*n* = 435)	3.38–3.84	3.48 ± 0.78	17.67 ± 2.91	17.40–17.95	52.14 ± 8.50	51.08–53.28
Living environment	Z = 3.527; *p* < 0.001 *		Z = 2.902; *p* = 0.004 *		Z = 1.253; *p* = 0.210	
Home (*n* = 359)	3.66 ± 0.72	3.56–3.77	17.44 ± 2.79	17.11–17.76	51.80 ± 8.82	50.53–52.99
Retirement home (*n* = 273)	3.33 ± 0.79	3.20–3.45	18.34 ± 2.67	18.05–18.64	50.49 ± 8.01	49.28–51.08
Education	H(3) = 3.305; *p* = 0.347		H(3) = 23.987; *p* < 0.001 *		H(3) = 16.324; *p* < 0.001 *	
Elementary	3.49 ± 0.76	3.30–3.67	18.03 ± 2.41	17.61–18.48	50.76 ± 8.47	48.48–52.88
Secondary	3.51 ± 0.75	3.42–3.62	18.00 ± 2.91	17.68–18.26	50.62 ± 7–88	49.54–51.70
Undergraduate	3.52 ± 0.85	3.30–3.73	18.07 ± 1.71	17.70–18.40	51.79 ± 10.03	49.07–52.50
Postgraduate	3.97 ± 0.83	3.37–4.58	18.11 ± 3.89	17.80–19.30	52.05 ± 9.05	50.03–54.82
Contacts with relatives/loved ones	H(3) = 31.420; *p* < 0.001 *		H(3) = 2.013; *p* = 0.570		H(3) = 3.510; *p* = 0.319	
Every day (*n* = 194)	3.60 ± 0.73	3.46–3.73	17.92 ± 2.22	17.60–18.25	51.55 ± 9.32	49.88–53.31
Several times per week (*n* = 135)	3.78 ± 0.66	3.65–3.92	17.76 ± 3.42	17.19–18.35	52.11 ± 8.78	50.29–53.88
Several times a month (*n* = 234)	3.24 ± 0.73	3.12–3.38	18.05 ± 3.07	17.65–18.46	49.92 ± 7.60	48.58–51.31
Once a month or less (*n* = 69)	3.35 ± 0.97	3.02–3.87	17.99 ± 0.97	17.65–18.14	50.80 ± 7.66	48.07–52.23

Note: Post-hoc pairwise comparisons following significant Kruskal–Wallis tests were conducted using Dunn’s test with Bonferroni correction. *p*—a significance value; *p* < 0.05 (*); H—Kruskal–Wallis test statistic; x—mean; CI—confidence interval; S—standard deviation; Z—Mann–Whitney U.

**Table 3:** Relationship between person-centred care, general health, psychological well-being, number of contacts with relatives or loved ones, and monthly income.

Variables	GH	PWB	PCC	CWR	Age	MI
General health (GH)	1	−0.036	−0.067	0.038	0.107 *	0.262 **
Psychological well-being (PWB)	−0.036	1	0.122 *	0.016	0.021	0.236 **
Person-centred care (PCC)	−0.067	0.122 *	1	−0.117 *	−0.127 *	0.036
Contacts with relatives/loved ones (CWR)	−0.038	0.016	−0.117 *	1	−0.052	0.134 *
Age	0.107 *	−0.021	−0.127 *	−0.52	1	−0.053
Monthly income (MI)	0.262 **	0.236 **	0.036	0.134 *	−0.053	1

Note: *p* < 0.05 (*), *p* < 0.01 (**).

**Table 4:** Regression analysis for the general health of older adults.

	Variables	B	SE	β	t	*p*
R^2^ = 0.219	Gender	0.785	0.265	0.121	2.964	0.005 *
	Age	0.785	0.281	0.141	3.131	0.002 *
	Person-centred care	−0.529	0.155	−0.140	−3.402	<0.001 *
	Contacts with relatives/loved ones	−0.143	0.136	−0.044	−1.056	0.291
	Monthly income	−0.142	0.089	−0.071	−1.599	0.110

Note: R^2^—coefficient of determination; B—unstandardised regression coefficient; SE—standard error; β—standardised regression coefficient; t—a value of t-statistic; *p*—a significance value; *p* < 0.05 (*); standard multiple regression (Enter method) was conducted. Predictors were selected based on theoretical relevance and significant bivariate correlations with psychological well-being.

**Table 5:** Regression analysis for psychological well-being of older adults.

	Variables	B	SE	β	t	*p*
R^2^ = 0.292	Gender	−0.736	0.781	−0.039	−0.942	0.347
	Age	1.481	0.430	0.136	1.990	0.038 *
	Person-centred care	1.401	0.554	0.122	2.228	0.017 *
	General health	−0.308	0.162	−0.109	1.882	0.055
	Contacts with relatives/loved ones	0.329	0.398	0.034	0.828	0.408
	Monthly income	1.682	0.316	0.212	5.472	<0.001 *

Note: R^2^—coefficient of determination; B—unstandardised regression coefficient; SE—standard error; β—standardised regression coefficient; t—a value of t-statistic; *p*—a significance value; *p* < 0.05 (*); standard multiple regression (Enter method) was used. Predictors were selected based on theoretical relevance and significant bivariate correlations with psychological well-being.

## Data Availability

Data are contained within the article.
